# Use of Adaptive Support Ventilation (ASV) in Ventilator Associated Pneumonia (VAP) - A Case Report

**Published:** 2009-06

**Authors:** Bipphy Kath, N Hemanth, Prashanti Marella, M H Rao

**Affiliations:** 1,2,3 Assistant Professor; 4 Dean & HOD

**Keywords:** Adaptive support ventilation, Ventilator associated pneumonia, Nosocomial infection

## Abstract

**Summary:**

Prolonged ventilation leads to a higher incidence of ventilator associated pneumonia(VAP) resulting in ventilator dependency, increased costs and subsequent weaning failures. Prevention and aggressive treatment of VAP along with patient friendly newer modes of ventilation like adaptive support ventilation go a long way in successful management of these cases.

## Introduction

Ventilator associated pneumonia (VAP) in the recent past has been threatening to be the most common and catastrophic nosocomial infection attributing to significant mortality and morbidity with severe impact on the healthcare costs incurred by the society^1^. VAP presents to the clinician along with diagnostic dilemmas, difficulties in the ventilatory management of the patient as the source of the disease is the ventilator itself! We present a case report of successful management of VAP by using adaptive support ventilation (ASV), a newer novel mode of ventilation known to increase patient ventilator synchrony in a cost effective manner^2^.

## Case report

**Fig 1:**
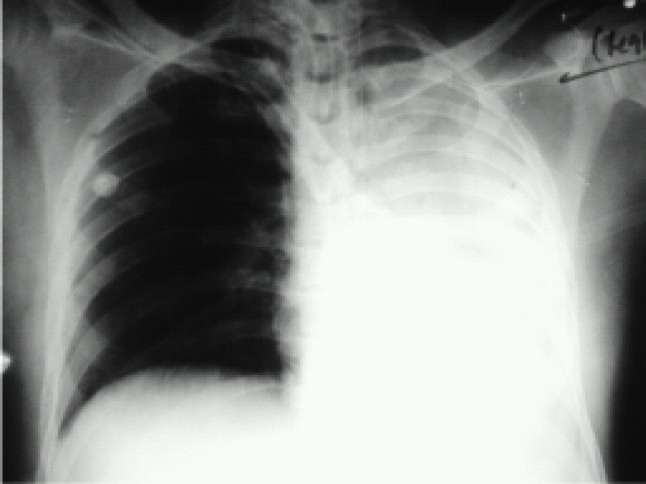
Chest X-ray on 6th day of ventilation showing VAP

A 35-year-old man was admitted in emergency with head injury following road traffic accident. Patient presented with left ear bleed, nasal bleed and unconsciousness with GCS 3. Endotracheal intubation was performed and CT scan revealed right fronto-temporoparietal acute sub dural haematoma and left parietal extradural haematoma with midline shift. Emergency craniotomy and evacuation was done and patient was electively ventilated for the next 24 hours. Post operative CT scan showed diffuse axonal injury (DAI) with poor neurological recovery. Day 2 onwards patient required higher FiO_2_ and so weaning was deferred (Table 1). Elective tracheostomy was done on the 4^th^ post operative day. Day 6 chest x-ray revealed consolidation of left middle and lower lobes (Fig 1) with deteriorating PaO_2_/FiO_2_ (P/F) ratios (Table 1). Provisional diagnosis of ventilator associated pneumonia (VAP) was made with the Clinical Pulmonary Infection Score (CPIS)^3^ of 7.

**Table 1:** Mode of ventilation and P/F ratios

Post Operative day	Mode of ventilation	PaO_2_	FiO_2_	P/F ratio
1	CMV	146	50	292
4	SIMV+PS	116	70	165
6	SIMV+PS	104	80	130
10	ASV (100% MV)	110	80	137
14	ASV (70% MV)	148	60	246
18	ASV (30% MV)	136	40	340

Patient was ventilated with volume controlled ventilation with high inspired oxygen concentration and PEEP. Endotracheal aspirates were sent for culture and sensitivity. Tracheal aspirate showed gram positive cocci in clusters and occasional gram negative bacilli. Broad spectrum antibiotics were already started. Rigorous chest physiotherapy was instituted. Culture reports confirmed the growth of Pseudomonas aeruginosa and Staphylococcus aureus. Appropriate antibiotics were administered according to culture sensitivity.

**Fig 2:**
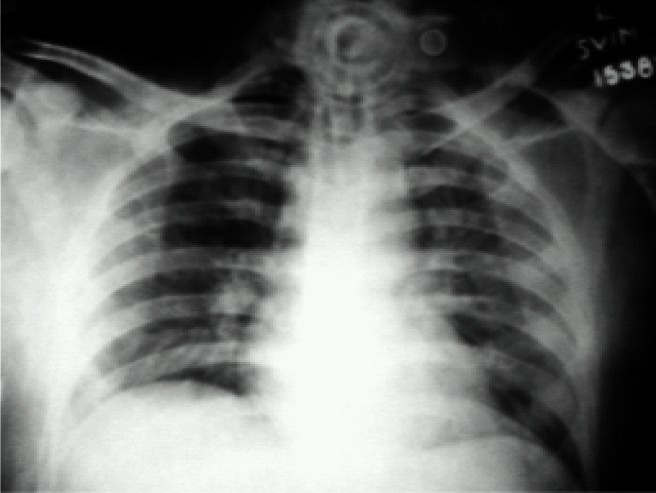
Chest X-ray on 14th day showing clearing of lung fields

**Fig 3:**
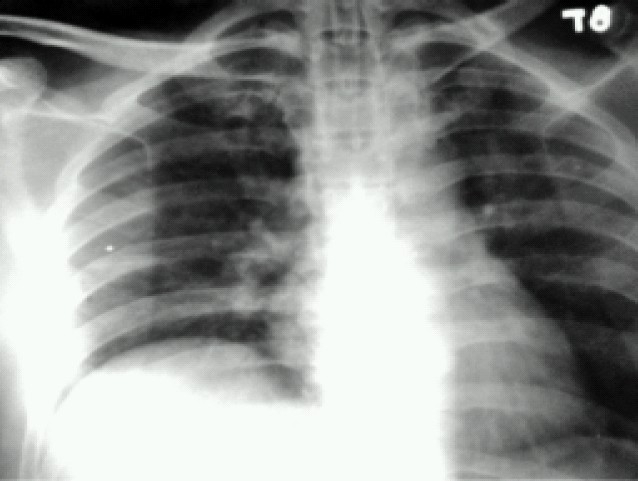
Chest X-ray on 19th day showing clear lung fields

Weaning was attempted over the next 4 days on SIMV (12 breaths) +Pressure support (15cm H_2_O) but failed as seen by worsening of blood gases. On the 10^th^ postoperative day mode of ventilation was changed to Adaptive support ventilation (Galileo ventilator by Hamilton medical systems). Minute volume was supported 100% to start with and the response was monitored by clinical improvement and arterial blood gases. Over the next 4 days oxygenation improved (P/F ratios) (Table 1). Support on minute volume was reduced in decrements of 10%. Chest physiotherapy, antibiotics and supportive measures (enteral feeds) were continued. The 14^th^ day chest x-ray showed clearing of lung zones (Fig 2). On the 15^th^ day patient was on minimal ASV support (FiO2< 0.4, PEEP = 5cm of H2O, minute ventilation supported at 30%). Spontaneous breathing trial was given over the next few days by alternating between ASV and T-piece initially in the ratio of 2:1, followed by 1:1 and then 1:2. On day 19, patient was weaned off the ventilator and shifted out of Neurosurgical ICU on 21st day (Fig 3)

## Discussion

Based on data from the National Nosocomial Infection Surveillance System, VAP represents the most common nosocomial infection seen in the intensive care unit (ICU). The incidence of VAP varies from 10% to 30% and crude mortality rates in VAP exceed 50%^4^. The attributable costs of VAP approach $20,000 as per western literature. A joint committee of the American Thoracic society (ATS) and Infectious Diseases Society of America (IDSA) has defined the various terms associated with VAP. VAP refers to pneumonia that arises more than 48–72 hours after endotracheal intubation^4^. Hospital acquired pneumonia (HAP) is defined as pneumonia that occurs 48 hours or more after admission, which was not incubating at the time of admission. Healthcare-Associated Pneumonia (HCAP) includes any patient who was hospitalized in an acute care hospital for two or more days within 90 days of the infection^4^. VAP is caused by a diverse range of pathogens. The microbiological profile of VAP varies across ICUs and hospitals. Pseudomonas aeruginosa and Staphylococcus aureus occur with similar frequency and when pooled account for nearly 30% of all cases of VAP^4^. VAP is typically categorized as early-onset VAP (occurring in the first 3-4 days of mechanical ventilation) and late-onset VAP (> 5 days). Early-onset VAP is commonly caused by antibiotic-sensitive community-acquired organisms (e.g., Streptococcus pneumoniae, Haemophilus influenzae, and Staphylococcus aureus). Late-onset VAP is commonly caused by antibiotic-resistant nosocomial organisms (e.g., Pseudomonas aeruginosa, methicillin-resistant Staphylococcus aureus, Acinetobacter species, and Enterobacter species)^4^.

VAP develops from aspiration of oropharyngeal secretions containing potentially pathogenic organisms, aspiration of gastric secretions and mechanical ventilation a major risk factor. Pugin and colleagues developed Clinical Pulmonary Infection Score (CPIS) as a diagnostic tool for pneumonia^3^. Mean score for patients with confirmed VAP was 6.5 for unconfirmed cases CPIS was 5.9.

The laboratory diagnosis for confirmation includes microbiological examination of endotracheal aspirate (EA) specimen, Blind protected telescoping catheter (PTC) sampling and BAL (bronchoalveolar lavage)^5^. Difficult weaning is a term reserved for patients who fail initial weaning and require up to three spontaneous breathing trials (SBT) or as long as 7 days from the first SBT to achieve successful weaning^6^. Sixth International Consensus Conference on Intensive Care Medicine stated that pressure support or assist–control ventilation modes should be favored in patients failing initial trial/trials.

Adaptive support ventilation (ASV) is a microprocessor-controlled mode (closed loop ventilation) of mechanical ventilation that maintains a predefined minute ventilation and an optimal breathing pattern (tidal volume and rate) by automatically adapting inspiratory pressure and ventilator rate to changes in the patient's condition^1^. When ASV was used on passive patients with different respiratory system mechanics (normal lungs, restrictive disease, or obstructive disease), the ventilatory pattern applied by the automatic controller was markedly different: frequency and I:E ratio were adapted as expected, according to the type and severity of the respiratory disease^1^. Adaptive Support Ventilation has been studied as the sole mode of ventilatory support in chronically ventilated patients and has been shown to be cost effective with minimal settings required to be altered for accomplishment of effective weaning process^7^. ASV has the capability to adjust automatically to the patients ventilatory requirements by selecting different VT-RR combinations based on respiratory mechanics in passive, mechanically ventilated patients^8^ and hence can be successfully applied to any subset of patients. The improved respiratory mechanics even allow for fast tracheal extubations in post cardiac surgery patients^9^. To conclude, VAP is a fatal nosocomial infection which besides antibiotic cover and supportive measures requires lung protective advanced mode of ventilation which can be provided by effectively by ASV.
